# Supplementary figures and images for: Adaptation of the World Health Organization Electronic Mental Health Gap Action Programme Intervention Guide App for Mobile Devices in Nepal and Nigeria: Protocol for a Feasibility Cluster Randomized Controlled Trial
Source: JMIR Res Protoc. 2021 Jun 15;10(6):e24115. doi: 10.2196/24115 (PMC8277329; doi:10.2196/24115)

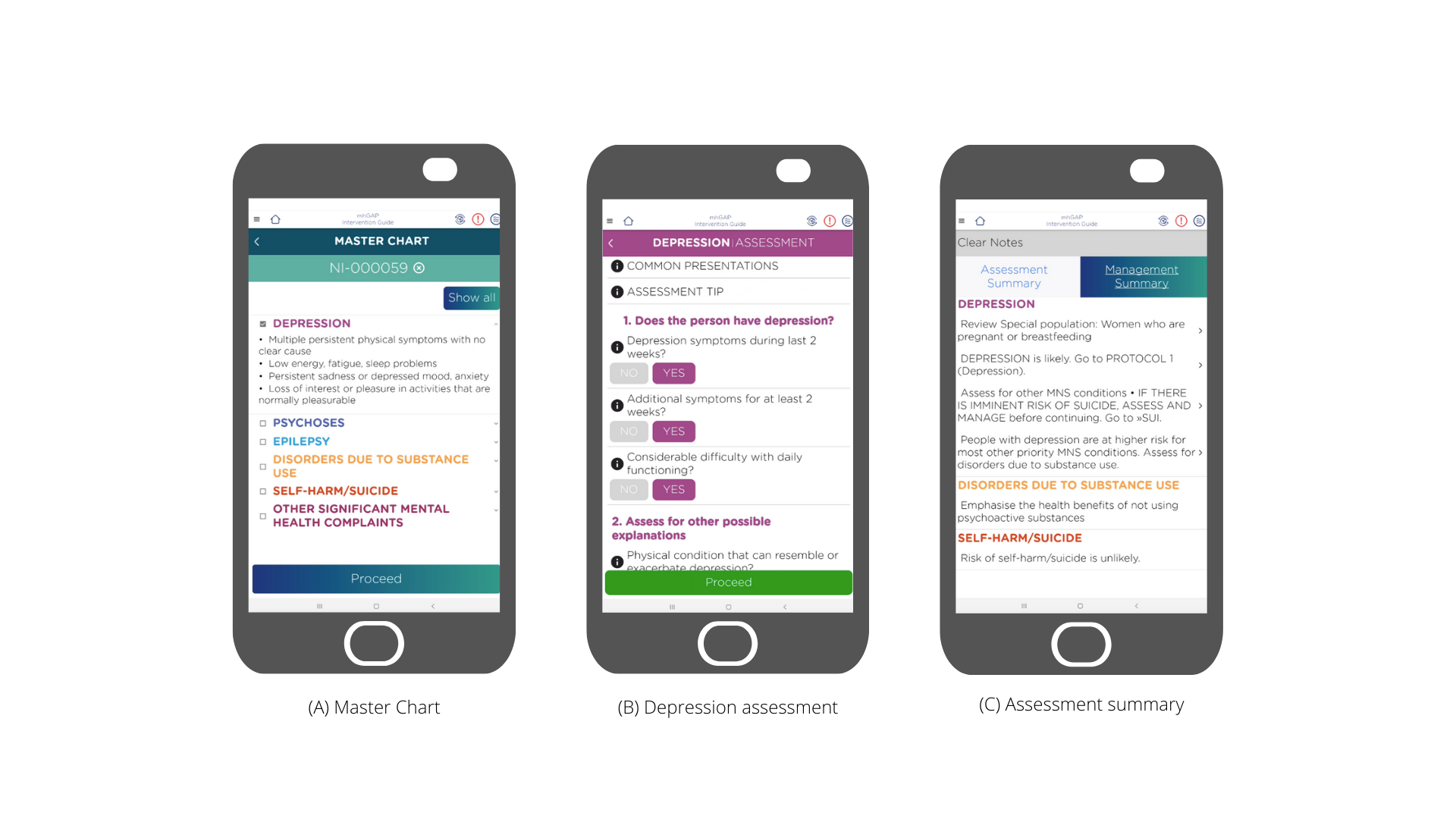

Supplement: Multimedia Appendix 1 [file resprot_v10i6e24115_app1.png]

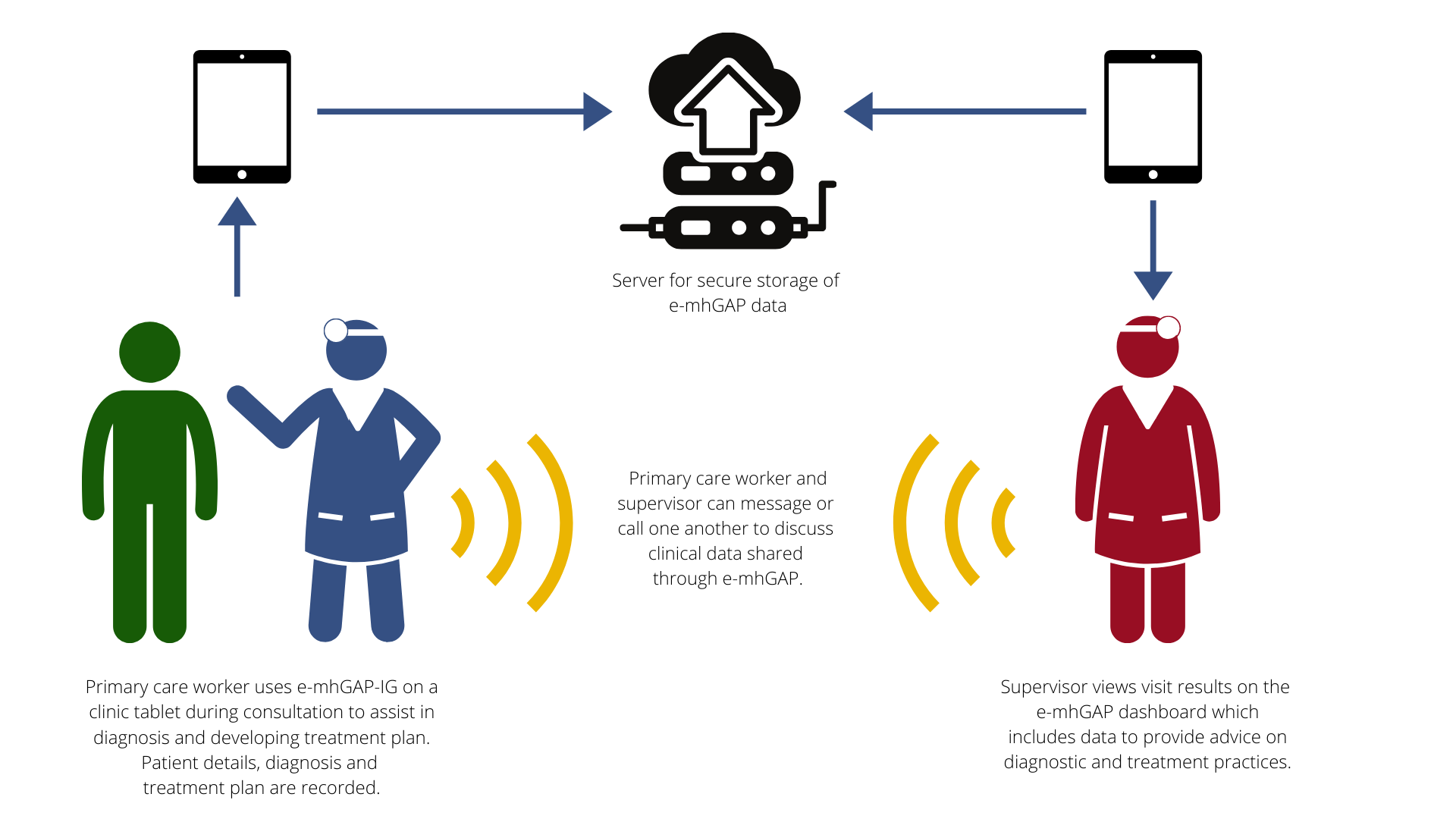

Supplement: Multimedia Appendix 2 [file resprot_v10i6e24115_app2.png]
