# Supplementary material for: Adaptation of the World Health Organization Electronic Mental Health Gap Action Programme Intervention Guide App for Mobile Devices in Nepal and Nigeria: Protocol for a Feasibility Cluster Randomized Controlled Trial
Source: JMIR Res Protoc. 2021 Jun 15;10(6):e24115. doi: 10.2196/24115 (PMC8277329; doi:10.2196/24115)
Supplement: Multimedia Appendix 3 [file resprot_v10i6e24115_app3.docx]

|  | **STUDY PERIOD** | | | | | | | | |
| --- | --- | --- | --- | --- | --- | --- | --- | --- | --- |
| **PRIMARY CARE WORKERS** (*direct beneficiaries*) – trained to deliver mental health services | | | | | | | | | |
|  | **Cluster Allocation** | **Enrolment** | **Post-allocation** | | | | | **Close-out** |  |
| **TIMEPOINT** | ***-t_1_*** | ***t_0_*** | ***t_1_ (PCW_0_)*** | ***t_2_ (PCW_1_, S_0_)*** | ***t_3_ (PCW_2_)*** | ***t_4_*** | ***t_5_ (PCW_3_, S_1_)*** | ***t_6_*** |  |
| **ENROLLMENT:** |  | | | | | | | | |
| Allocation | X |  |  |  |  |  |  |  |  |
| Eligibility screen |  | X |  |  |  |  |  |  |  |
| Informed consent |  | X |  |  |  |  |  |  |  |
| **INTERVENTIONS:** |  | | | | | | | | |
| Paper mhGAP training and remote supervision (control) |  |  | ←−−−−−−−−−−−−−−−−−−−−−−−−−−→ | | | | |  |  |
| e-mhGAP training and remote supervision (experimental) |  |  | ←−−−−−−−−−−−−−−−−−−−−−−−−−−→ | | | | |  |  |
| **ASSESSMENTS:** |  | | | | | | | | |
| *mhGAP knowledge* |  |  | X | X | X |  | X |  |  |
| *Depression Attitude Questionnaire (R-DAQ)* |  |  | X | X | X |  | X |  |  |
| *Social Distance Scale (SDS)* |  |  | X | X | X |  | X |  |  |
| *Clinical Competency (ENACT)* |  |  | X | X | X |  | X |  |  |
| *Perceptions of Supervisory Support Scale (PSS)* |  |  |  |  |  |  | X |  |  |
| *Acceptability of Intervention Measure (AIM)* |  |  |  | X | X |  | X |  |  |
| *Feasibility of Intervention Measure (FIM)* |  |  |  | X | X |  | X |  |  |
| *Intervention Appropriateness Measure (IAM)* |  |  |  | X | X |  | X |  |  |
| *Organizational Readiness for Implementing Change (ORIC)* |  |  |  | X |  |  |  |  |  |
| *mhGAP fidelity* |  |  |  |  | X |  |  |  |  |
| *mhGAP adoption* |  |  |  | X |  |  | X |  |  |
| *mhGAP operational cost* |  |  |  |  | X |  |  |  |  |
| *Normalization Measure Development (NoMAD)* |  |  |  |  |  |  | X |  |  |
| *Qualitative interviews* |  |  |  |  |  |  | X |  |  |
| **PATIENTS** (*indirect beneficiaries*) – patients treated by primary care workers trained in paper mhGAP vs. e-mhGAP | | | | | | | | | |
|  | **Cluster Allocation** | **Pre-Training** | **Post-Training** | | | | | **Close-out** |  |
| **TIMEPOINT**** | ***-t_1_*** | ***t_0_ (P_0_,F_0_)*** | ***t_1_*** | ***t_2_*** | ***t_3_*** | ***t_4_ (P_1_,F_1_)*** | ***t_5_*** | ***t_6_ (P_2_)*** |  |
| **ENROLLMENT:** |  | | | | | | | | |
| Allocation | X |  |  |  |  |  |  |  |  |
| Eligibility screen |  |  |  |  |  | X |  |  |  |
| Informed consent |  |  |  |  |  | X |  |  |  |
| **INTERVENTIONS:** |  | | | | | | | | |
| mhGAP |  |  |  |  |  | ←−→ |  |  |  |
| **ASSESSMENTS:** |  | | | | | | | | |
| *Patient Health Questionnaire (PHQ-9)* |  | X |  |  |  | X |  | X |  |
| *World Health Organization Disability Assessment Schedule (WHODAS 2.0)* |  | X |  |  |  | X |  | X |  |
| *Health Worker Diagnosis (mhGAP)* |  | X |  |  |  | X |  | X |  |
| *Composite International Diagnostic Interview (CIDI)* |  | X |  |  |  | X |  | X |  |
| *Treatment initiation (clinical notes)* |  | X |  |  |  | X |  | X |  |
| *Therapuetic encounter (ENACT-Service user version)* |  |  |  |  |  | X |  | X |  |
| *Acceptability of Intervention Measure (AIM)* |  |  |  |  |  |  |  | X |  |
| *Intervention Appropriateness Measure (IAM)* |  |  |  |  |  |  |  | X |  |
| *Qualitative interviews* |  |  |  |  |  |  |  | X |  |
| Note: All primary care clinics (clusters) are allocated are -*t*_1_. Primary care workers are assigned to paper mhGAP training plus remote supervision (control arm) or e-mhGAP training plus remote supervision (experimental arm) trainings based on the primary care clinic in which they work. For one month prior to trainig, patients will be enrolled to assess accurate detection rates (t_0_). Primary care workers are enrolled and consent at *t*_0_. Primary care workers are administered assessment batteries immediately prior to training at *t*_1_. They then participate in training and subsequent supervision for the duration of the research study. There is an immediate post-training assessment at *t*_2_, followed by a 3-month (*t*_3_) and 8-month (*t*_5_) assessment. Close-out qualitative interviews are conducted with a subset of primary care workers and all supervisors at *t*_5_. Patients are enrolled with paper mhGAP or e-mhGAP trained primary care workers according to the allocation of their local primary health clinic. Patients diagnosed by primary care workers receive mhGAP-based interventions. Patient enrollment occurs at approximately 3 months after primary care workers are trained. Patients are assessed at treatment initiation (*t*_4_) and 3-months later at study close-out (*t*_6_). Close-out qualitative interviews are conducted with a subset of patients at *t*_6_. | | | | | | | | |  |
