# Supplementary material for: Adaptation of the World Health Organization Electronic Mental Health Gap Action Programme Intervention Guide App for Mobile Devices in Nepal and Nigeria: Protocol for a Feasibility Cluster Randomized Controlled Trial
Source: JMIR Res Protoc. 2021 Jun 15;10(6):e24115. doi: 10.2196/24115 (PMC8277329; doi:10.2196/24115)
Supplement: Multimedia Appendix 4 [file resprot_v10i6e24115_app4.pdf]

MRC Peer Reviews, EMILIA

proposal-ID	Research Quality	Research Environment and People	Impact	Ethics	Data Management	Resources Requested	Overall Assessment	result
GACD-272	1.This proposal makes the case for studying the feasibility of electronic version of the WHO supported mhGAP-IG compared to the paper version. This is potentially impactful on increasing access to trained personnel to deliver mental health care packages given the widening availability of mobile electronic devices in LMICs. Utilizing an electronic version of mhGAP-IG to train non-psychiatrists to deliver care for common mental health disorders as detailed in the application could be a cost effective way to make it more available in low resource setting like Nigeria and Nepal where use of electronic devices have exploded over the last 10 years.<br>2. This proposal includes innovative approaches to training, supervision and adaptation of the electronic mhGAP-IG<br>3. Detailed data sharing outlined appropriately<br>4. The research plans as outlined appear feasible, including appropriate methodology, design and preliminary data	1. The available records and their areas of expertise indicate that the investigators have the expertise and track record well suited to deliver on this proposal<br>2. They have institutional support that can enable this research<br>3. The facilities are available	1. Very high impact if successful. It will increase the pool of trained mental health providers by making mhGAP more accessible.<br>2. Potentially can improve access to mental health thus reducing the burden of disease attributable to common mental health disorders in Nigeria and Nepal.<br>3. The investigators have laid out detailed plan of training which seems sustainable	Details of their ethical review is satisfactory	Detailed Data management plan is satisfactory	Funds requested are essential and justified	4	4
GACD-272	The applicants have made a strong case and if successful is likely to lead to global impact.<br><br>It was not clear if the culturally adapted versions of the psychosocial interventions are available for both Nepal and Nigeria.<br><br>The PHQ-9 is being used as a self rating scale will the individuals who are not literate be excluded from the study?<br><br>Can the applicants provide any reference reference for assuming intraclass clustering of 0.02.<br><br>I am impressed by the focus on the development of early career research staff.<br><br>Probably because of their experience in this area applicants do not focus much on the risks.	The applicants are one of the strongest research teams in global mental health and have made a strong case for the rationale for the proposed research. The application is well written but I do have some queries.<br><br>The applicants should give the rationale for purposefully selecting Nepal and Nigeria.<br><br>Can the applicants clarify the role of KCL CTU I did not see any costings for the CTU.<br><br>It was very useful to have the country specific details.	The proposed study is proof of concept therefore will have limited impact though at places it was not very clear if this is a proof of concept study or effectiveness trial applicants say "scale up provision of evidence based mental health care for people with mental illness".	I have no major concerns but I will be surprised if there are no adverse events related or not related to the study. I suggest that the investigators also record the side effects of the psychosocial interventions both the paper versions and the M health one.<br><br>I am sure the ethics committees will request for a distress policy.<br><br>The applicants need to clarify further regarding support and supervision if there is risk of harm to self or others the current statement is quite vague.	The applicants have a sound plan for data management and have considered the information security	I could not find the total costs but over all the costs look ok. Investigator time and involvement related to project management is appropriate. It will be useful to have the details of £60300 WHO Software development costs how have these been calculated.<br><br>I could not understand such a big difference in resources for Nigeria and Nepal.	5	5

GACD272: Thornicroft et al Emilia project. Response to comments of Reviewers 1-2.

We thank Reviewer 1 for his/her wholly positive comments about the Emilia proposal. Regarding the points raised by Reviewer 2, we respond to each of these in turn.

1. It was not clear if the culturally adapted versions of the psychosocial interventions are available for both Nepal and Nigeria.
In ***Nepal,*** there has been a long track record of cultural adaptation of mental health interventions[1]. The process of cultural adaptation is rooted in psycho-ethnographic research conducted by one of the applications (BK). Furthermore, in recent years, through the PRIME[2] and Emerald [3] programmes, the Nepal team has developed a WHO mhGAP-based mental health care plan that has been tailor-made to the Nepali context[4,5]. This has involved a rigorous process of stakeholder and theory of change consultations[6], formative research and adaptation workshops[4]. Specifically for psychological treatments, we started by making cultural adaptations to the generic counselling approach that was developed by Transcultural Psychosocial Organization Nepal (TPO Nepal), an non-governmental organisation[1]. More recently, we have worked with psychological interventions that have been developed specifically in the South Asian setting, such as the Healthy Activity Program and Counselling for Alcohol Problems[7,8]. In ***Nigeria*** the WHO mhGAP Intervention Guide (mhGAP-IG) has also undergone comprehensive adaptation and contextualization. The exercise, consisting of structured engagement with potential users and workshops with stakeholder groups, including specialists in mental, neurological and substance use, primary health care workers, and facility managers produced a version suited to the local cultural and health system contexts. The adapted version has subsequently been used in a pilot demonstration project in a state in Nigeria[9-11].

2. The PHQ-9 is being used as a self-rating scale - will the individuals who are not literate be excluded from the study?
In ***Nepal***, the cultural adaptation and clinical validation of the PHQ-9 (performed by this applicant team) focused specifically on the feasibility and psychometrics when administered by non-specialists to populations with limited literacy in primary care settings. The rewording of questions and use of pictures for response options was undertaken to modify the PHQ-9 into a tool administered by auxiliary health workers or by researcher assistants. Therefore, this tool can now be used in Nepal without restriction to literate populations. The psychometric properties for the adapted and validated PHQ-9 in Nepal are comparable to the self-administered version in high-income countries: with a Nepali PHQ-9 cut-off ≥ 10: sensitivity = 0.94, specificity = 0.80, positive predictive value=0.42, negative predictive value = 0.99, positive likelihood ratio=4.62, and negative likelihood ratio = 0.07[12]. In ***Nigeria***, the PHQ9 will be self-administered by respondents with at least 10 years of formal education. Those with less education will have the questionnaire read to them by trained research assistants. There is substantial experience in implementing interviewer-administered screening in this way using the PHQ9 in Nigeria[13].

3. Can the applicants provide a reference for assuming intra-class clustering of 0.02?
The reference for assuming intra-class clustering of 0.02 is Adams et al[14].

4. Probably because of their experience the applicants do not focus much on risks.
As the applicant team does have very extensive experience in undertaking research projects in low income settings, we shall draw upon our protocols and procedures used in prior studies to set out a clear risk avoidance, mitigation and management plan at the outset of the project, if funded. ***Risk management of the consortium*** is led by KCL, which has used risk protocols (eg in PRIME, Emerald) in 6 low and middle income countries (LMICs). This plan will include provisions, e.g. for budget management, non-completion of milestones or deliverables, clear and fair rules for authorship of papers, an ethics and liability agreement, and a step-wise procedure for conflict resolution. Regarding the risk of insufficient scientific credit going to LMIC staff, we shall actively support the career and promotion needs of early

and intermediate career staff in particular e.g. by promoting equity in supporting these colleagues to be first authors on papers[15-17].

5. Applicants should give the rationale for purposefully selecting Nepal and Nigeria.
Nepal is a part of the Emerald and PRIME research consortia, which have worked for the past 5 years on the set-up, implementation and evaluation of the WHO mh-GAP-IG. As a result of this, a great deal of preparatory work has been completed in terms of materials development, but also in terms of the support from policy maker and service user groups[5,11]. As a result of this, the integration of mental health into primary health care is now recognized by the Nepal Ministry of Health. This has resulted in the adoption of the WHO mhGAP-based training curricula for the health workers, as well as the mh-GAP recommended psychotropic drugs being part of the free drugs list in Nepal. Further, TPO Nepal has developed a specialized mental health research infrastructure with over 120 staff in Nepal. In the aftermath of the devastating 2015 earthquakes, the WHO mhGAP-based mental health care plan was scaled up in 8 of the most affected districts[18]. In ***Nigeria*** the WHO mhGAP-IG has been contextualised to the country's health system and where the tool has been subjected to a comprehensive demonstration study and also used in a randomized controlled study[9,10]. In 2013, the Nigeria Council of Health, the highest health policy-making body in the country, adopted the WHO mhGAP Implementation Plan as the pathway to scaling up mental health service throughout Nigeria. The proposed project will provide empirical evidence for using readily available technological tool to enhance the programme implementation. For these reasons Nepal and Nigeria provide examples of low and middle income countries respectively which both have very substantial gaps in their service provision[19], and which show high levels of readiness (in both clinical and research terms) to assess the feasibility of the e-version of the mh-GAP app in primary health care settings.

6. Can applicants clarify the role of KCL CTU I did not see any costings for the CTU?
The Emilia project is a proof-of-concept project (and not an RCT) of the feasibility of implementing the e-version of the mhGAP IG. We shall use the Emilia project to inform the design and implementation of a future large scale RCT. We plan that the KCL CTU will be an integral part of the future RCT study. Therefore CTU trial costings are not included in the current bid. The medical statistician in the Emilia team (IB) is employed at the KCL CTU. (For the information of the panel: the KCL CTU was formed in 2002 and has core funding from both NIHR and KCL. Currently there are approximately 175 clinical trials sponsored or co-sponsored by the CTU. In 2016 the CTU gained more NIHR funded trials than any other registered CTU in the UK).

7. The proposed study is proof of concept therefore will have limited impact though at places it was not very clear if this is a proof of concept study or an effectiveness trial.
This is a proof of concept study. We intend to use its results to support the proposal for a subsequent effectiveness RCT.

8. I have no major concerns but I will be surprised if there are no adverse events related or not related to the study. I suggest that the investigators also record the side effects of the psychosocial interventions both the paper versions and the M health one. I am sure the ethics committees will request for a distress policy.
This is an important point. Within ***TPO Nepal,*** we have a detailed Adverse Events Reporting Mechanism, which is overseen by a Data and Safety Monitoring Board. The use of a e-version of the mGAP IG will actually facilitate the reporting of side effects and (serious) adverse events, because common and serious side effects and adverse reactions to both pharmacological and psychosocial treatments can be systematically evaluated and monitored, thus triggering a response protocol. Therefore, an advantage of the e-version of mhGAP is the ability to better systematically record, monitor, and respond to adverse events. Regarding a distress policy, this will be closely based upon such policies that we are currently using in LMICS in similar studies (PRIME, Emerald, COBALT). While the mhGAP IG includes psychosocial interventions such as psycho-education and problem

solving therapy, which we anticipate will diminish distress, there is a potential risk of brief or transitory distress when discussing troubling issues in the person's life, as well as clinical deterioration despite treatment. The healthcare providers will be trained to detect this at each assessment using pre-defined protocols, using the available referral pathways to ensure appropriate care. In addition, healthcare provider staff will be trained to use the mhGAP IG app in a way that does not interfere with empathic communication and care. ***Serious Adverse Events*** will be defined as any untoward medical occurrence that results in death, requires hospitalization or causes significant or persistent incapacity/disability, or in the opinion of the investigators represents other potentially significant harm to research subjects. Based on our previous experiences with studies of depression in Nepal and Nigeria, we will monitor three types of serious adverse events (suicide attempts, hospitalization and death), and healthcare providers will follow a pre-defined referral protocol. We are familiar with **adverse event reporting** requirements for MRC Guidelines for Management of Global Health Trials and we shall fully comply with these.

10. The applicants need to clarify further regarding support and supervision if there is risk of harm to self or others the current statement is quite vague.
In ***Nepal***, there are multiple tiers of support and supervision in place. In the district where the study will be implemented, there will be TPO Nepal staff on site during the study to whom primary care workers, patients, and family can refer any concerns regarding clinical or social adverse outcomes. In addition, TPO Nepal employs a full-time psychiatrist who is available 24-hours per day for emergencies. In addition, as with prior studies, TPO Nepal develops a referral pathway with the closest psychiatric specialist to the study site to receive referrals. In the case of suicidality and other forms of self-harm, TPO Nepal has developed a 4-step protocol which is used in all studies. In ***Nigeria*** there will be two levels of supervision. The first level will be provided by general physicians who are the supervisory clinicians for workers in the primary health care clinics. Second, these doctors will be able to consult with and refer to psychiatrists at the University College Hospital in Ibadan.

11. It will be useful to have the details of £60,300 WHO Software development costs how have these been calculated.
These **c**osts have been calculated based on WHO's experience of developing several prior e-health interventions. The total cost (£60,300) will be divided between: (a) technology enhancement for the Remote Supervision and Support module; (b) iterative refinement of the platform with user testing and feedback; and (c) translation into the local languages.

12. I could not understand such a big difference in resources for Nigeria and Nepal.
The difference between the site costs, including the resource allocation, is based upon actual local costs of salaries in Nigeria and Nepal. The budget has been carefully costed to ensure that all sites are sufficiently funded to achieve the study objectives.

References
1. Tol WA et al Transcultural Psychiatry 2005; 42: 317-33. 2. Semrau M, et al.. BMC Med 2015; 13: 79. 3. Lund C. PLoS Med 2012; 9(12): e1001359. 4. Jordans MJD, British Journal of Psychiatry 2016; 208(s56): s21-s8. 5. Gurung D, et al. Int J Ment Health Syst 2017; 11: 30. 6. De Silva MJ, et al. Trials 2014; 15: 267. 7. Patel V. et al. Lancet 2017; 389(10065): 176-85. 8. Nadkarni A. et al. Lancet 2017; 389(10065): 186-95. 9. Abdulmalik J. et al. PLoS Med 2013; 10(8): e1001501. 10. Gureje O. et al. BMC Health Serv Res 2015; 15: 242. 11. Mugisha J. et al. Int J Ment Health Syst 2017; 11: 7. 12. Kohrt BA. et al. BMC Psychiatry 2016; 16: 58. 13. Obadeji A. et al. J Family Med Prim Care 2015; 4(1): 30-4.14. Adams G. et al. J Clin Epidemiol 2004; 57(8): 785-94. 15. Selamu M. et al.. PLoS One 2015; 10(5): e0126666. 16. Upadhaya N. et al. Int J Ment Health Syst 2016; 10: 60. 17. Thornicroft G. et al. Harv Rev Psychiatry 2012; 20(1): 13-24. 18. Kane JC et al, Epidemiol Psychiatr Sci 2017: 1-10. 19. Thornicroft G. et al. British Journal of Psychiatry 2017; 210(2): 119-24.
